# Physical examination performed by general practitioners in 5 community health service institutions in Beijing: an observational study

**DOI:** 10.1186/s12875-021-01619-1

**Published:** 2022-01-14

**Authors:** Yun Wei, Feiyue Wang, Zhaolu Pan, Meirong Wang, Guanghui Jin, Xiaoqin Lu

**Affiliations:** 1 Department of General Practice, Beijing Tongren Hospital, Capital Medical University, Beijing, China; 2 Department of General Practice, School of General Practice and Continuing Education, Capital Medical University, No. 10, Xitoutiao, You’anmenwai, Fengtai District, Beijing, 100069 China

**Keywords:** Physical examination, Consultation, Community health service, General practitioner, Beijing

## Abstract

**Background:**

Physical examination is a core component of consultation. Little is known about the status quo of physical examinations performed by general practitioners in community health service institutions in China. The aim of this study was to investigate general practitioners’ performance of physical examinations in consultations.

**Methods:**

An observational study was conducted in 5 community health service institutions in Beijing between November 2019 and January 2020. Eleven general practitioners were observed for one workday. Information of consecutive consultations was recorded including patient characteristics, reasons for encounter, physical examinations performed by general practitioners, length of consultation time and time spent on specific activities in consultations.

**Results:**

A total of 682 consultations of 11 general practitioners were recorded. Physical examinations were performed in 126 consultations (15.8%). Physical examination was more likely to be performed in patients visiting with symptoms (*P* < 0.001). Majority of the 126 physical examinations were distributed in “Head, face, and neck examination” (*n* = 54, 42.9%) and “Cardiovascular examination” (*n* = 55, 43*.*7%). No physical examination was performed on skin, male genitalia, female breasts and genitalia, and neurological systems. Total 2823 min of activities were observed and recorded. General practitioners only spent 3.1% of the recorded time on physical examination, which was less than the time spent on taking history (18.2%), test (4.9%), diagnosis (22.7%), therapy (38.4%), and health education (8.6%). The average time spent on physical examinations was 0.8±0.4 min per consultation.

**Conclusion:**

Physical examination was insufficiently performed by general practitioners in community health service institutions in Beijing. More time and commitment should be advocated for improving the quality of physical examinations in primary care.

**Supplementary Information:**

The online version contains supplementary material available at 10.1186/s12875-021-01619-1.

## Background

Primary care is an essential component of high-performing health care system. Due to the dilapidated primary care infrastructure and health care inequity, promoting community health service became a prioritized agenda in the health care reform of China [1]. Primary care network was re-strengthened based on the development of community health service institutions (CHSIs) in China, comprising community health centers (CHCs) and community health stations (CHSs). CHSs are small-scale clinics as satellite sites of CHCs [2]. General practitioners (GPs) are the first contact of health care in CHCs and CHSs delivering ambulatory care for patients with acute and chronic diseases in community [3]. Till 2019, there were 35,013 CHSIs across China, and the number of patient visits to CHSIs was 860 million in 2019 [4], which was over twice the number in 2009 [5].

Physical examination (PE) is a key process for medical diagnosis and a core component of consultation [6], which obtains findings via inspection, palpation, percussion, and auscultation [7]. Previous evidence indicated that obtaining a good patient history could result in correct diagnosis in 70% of cases, with a thorough physical examination, the rate would increase to 90% [8]. Performing PE in general practice not only helps to collect diagnostic information but expresses the fundamental humanity of doctor-patient relationships [9]. Doctors’ patience and physical contact may relax patients and make them feel cared. It is essential for providing patient-centered care, which is a fundamental principle of general practice [10].

However, with the expansion of laboratory tests, there is a tendency for clinicians relying increasingly on laboratory reports rather than PE and clinical judgment [11–13]. It was reported in the United States that physicians spent less than 18% of their on-duty time on PE in patients admitted to hospital service [14]. Inadequacies of physical examination may influence patient care. As indicated in a study, almost 50% of diagnostic errors found in outpatient clinics can be traced to errors in PE [15]. The errors in most cases were that the appropriate PE maneuver has never been performed in consultations [16].

Considering the trend that clinical use of PE in hospital has decreased, there is a concern about the adequacy of GPs’ performance of PE in general practice consultations [11–14]. A study in Australian general practice reported that PE was observed in 64.5% of consultations and another study in Estonia showed that PE was performed in 79.0% of consultations [7, 17]. As the number of visits to CHSIs is increasing very fast, the performance and quality of PE, in appropriate diagnosis and management could be undermined. However, the GPs’ performance of PE was inconsistently reported in China. A study in Guangzhou reported 72.8% of consultations involved PE [18], while another study in Beijing found that PE only took place in 28.0% of consultations [19]. These studies reported the performance rate of PE, however, detailed information of PE in general practice consultations has not been depicted yet in China. Thus, this study aimed to investigate detailed information of the performance of PE in general practice consultations and explore possible differences in PE across GP characteristics, to provide evidence for improving the quality of general practice consultations in China.

## Methods

This was an observational study conducted in five CHSIs in Beijing, China between November 2019 to January 2020.

### Ethics statement

This study was approved by the Ethical Committee of the Capital Medical University, Beijing, China. Written informed consent was obtained from each participating GP in this study. Verbal consent was obtained from patient because acquisition of written consent could potentially interfere the consultation process. All participants’ information was kept confidential and tracked anonymously with identification number only.

### Setting and participants

The study was conducted in five CHSIs in Beijing as a convenience sample with ensured accessibility and availability for patients and stable amounts of visits. Purposive sampling was used to recruit GPs according to the following criteria: (a) work experience in general practice for over 2 years; (b) stable amounts of visits; (c) consent to participate in this research. GPs who were seeing patients for only half a day per week and GPs who were trainees rotating in CHCs/CHSs were excluded. Fifteen eligible GPs were invited, and eleven GPs agreed to participate in the study. All consecutive patients visiting the recruited GPs on the workday of observation were recruited with verbal consents. Patients were excluded if they visited for illness certificate or didn't register for a formal consultation.

### Observation form

The observation form in this study was based on the Municipal Medical Regulations on CHSIs in Beijing and previous literatures [17–20]. Prior to the study, two GPs were observed to test and modify the observation form. The modified observation form consisted of patient characteristics (age, sex, insurance status, etc), reasons for encounter (RFEs), PEs performed by GPs, the length of consultation time and time spent on specific activities (including history taking, PE, test, diagnosis, therapy, and etc.) performed by GPs.

### Data collection

Each GP was observed for one workday during November 2019 to January 2020. The observation was between the beginning and end of workday (from 8 am to 5 pm), excluding time spent in none-consultation activities (e.g. lunch, meeting).

Three postgraduate students (one full-time master candidate and two PhD candidates in general practice) were trained as observers in this study. A training session was conducted before the observation. Medical activities performed by GPs and time spent in consultations were recorded by the observer. For general examination, the observer would record the activities visibly performed in GP-patient consultations, such as temperature and lymph node examination. The activities of inspection, such as first impression, nutritional status, mental state examination (MSE), were not recorded due to the difficulty in identifying the activities by the observer. For skin examination, the observer would record the activities if the GPs made obvious inspection or asked patients to expose skin. Comprehensive PE was defined as thorough examination of multiple systems of the whole body in this study. The length of consultation was recorded with phone timer which was from the patient sitting down till the patient leaving the consultation room. Specific PE examinations and time spent on consultation activities were recorded. When multiple activities were performed at the same time, all activities were recorded in the same interval. Given that checking the notes with GPs after each consultation will potentially affect workflow, the information recorded in consultations was checked with GPs at the end of one-day observation. To avoid interrupting GPs’ performance, we would explain to GPs that this research will not affect their annual performance appraisal. For each GP, there was an observer (master candidate or PhD candidate) seated in the least intrusive corner of consultation room who would talk to neither the GPs nor patients. Additionally, information of the participated GPs was collected, including age, sex, education, working years, professional position, and training experience.

### Data coding

The RFEs were coded using the International Classification of Primary Care, second edition (ICPC-2), which is commonly used in primary care settings [21, 22]. This standardized classification is based on codes that are classified in 17 chapters representing body systems and problem areas [23].

### Statistical analysis

Descriptive analyses were used to describe the characteristics of patients, GPs and medical activities. Means [with standard deviation (SD)] were used to report continuous variables, while frequencies (%) were used to report categorical variables. Differences between groups were tested using chi-square test. A 2-tailed *P* < 0.05 was regarded as statistically significant. The minutes tallied for each activity were manually abstracted, for which summary statistics were converted to percentage of total minutes. Data management and analyses were performed using Statistical Package for Social Science (SPSS), version 22.0.

## Results

### Characteristics of GPs

Eleven GPs participated in this study and the mean age (with SD) was 39.4±4.3 years. Nine GPs were female. Ten GPs had a bachelor’s degree and nine GPs had over 10 years of work experience. There were six GPs with senior grade title, four GPs with intermediate grade title and one GP with junior grade title. Nine GPs had training experience in general practice, including three GPs in standardized residency training program and six GPs in on-job training program. The average number of patient visits for each GP on one workday was 62.0±13.6 (ranged from 41 to 88). Three GPs saw more than 70 patients, six GPs saw 51-70 patients, and two GPs saw less than 50 patients on the observed workday (Table 1).

**Table 1:** Demographic characteristics of participated GPs (*n* = 11)

Characteristics	Frequency	Percentage (%)
Institution		
CHC	9	81.8
CHS	2	18.2
Sex		
Male	2	18.2
Female	9	81.8
Education		
Bachelor’s degree	10	90.9
Master’s degree	1	9.1
Working years		
≤10	2	18.2
>10	9	81.8
Professional title^a^		
Junior grade title	1	9.1
Middle grade title	4	36.4
Senior grade title	6	54.5
GP training^b^		
Standardized residency training	3	27.3
On-job training	6	54.5
No training experience	2	18.2
Patient volume on the observation unit		
≤50	2	18.2
51-70	6	54.5
>70	3	27.3

Abbreviation: *GP* general practitioner, *CHC* community health centre, *CHS* community health station

^a^In China, professional titles in medicine include junior grade, middle grade, and senior grade titles, which are based upon work experience and research achievement of health professionals

^b^Since 2011, GPs were trained through three programs in China: (i) the standardized residency training (3-year residency training for graduates of 5-year medical school study), (ii) on-job training (1-year training for doctors who want to register as GP), (iii) assistant GP training (2-year training for graduates of 3-year junior medical college study). Assistant GP after training always work as a rural GP in China. Some GPs who started working in primary care institutions before 2011 have no training experience

### Characteristics of Patients

A total of 682 consultations were observed in this study. Among all the patients, 53.1% were female. The mean age (with SD) of patients was 61.9±14.4 years. The age distribution of all patients (with 2 missing) was 2.1%, 9.8%, 44.6% and 43.3% for those aged 25 years or less, 26 years to 45 years, 46 years to 65 years and over 65 years, respectively. Majority of the patients (96.2%) were covered by basic medical insurance, 0.1% of the patients had business insurance, and only 2.9% of the patients had no medical insurance. There were 29.9% of patients with only one health problem, 26.0% of patients with two health problems, and 44.1% of patients with three or more health problems (Table 2).

**Table 2:** Demographic characteristics of patients in the study (*n* = 682)

Characteristics	Frequency	Percentage (%)
Sex		
Male	320	46.9
Female	362	53.1
Age (years)		
≤25	14	2.1
26-45	67	9.8
46-65	304	44.6
>65	295	43.3
Missing	2	0.3
Social medical insurance		
Basic medical insurance	656	96.2
Business insurance	1	0.1
Other insurance	5	0.7
Without medical insurance	20	2.9
Number of health problems^a^ discussed		
1	204	29.9
2	177	26.0
3 or over	301	44.1

Note: ^a^health problems here refer to GPs’ diagnoses of patient’s disease

### Patients’ reasons for encounter

There were 1608 RFEs (2.4 per encounter) recorded from 682 consultations. Among all the RFEs, 685 were new symptoms for encounter, 850 were prior chronic conditions, and 73 RFEs were from patients visiting for test and therapeutic consultation. The top three RFES of patients with new symptoms were “R5 cough” (*n* = 111, 16.2%), “R21 throat symptoms” (*n* = 89, 13.0%) and “R25 sputum/sputum abnormalities” (*n* = 66, 9.6%). The top three health problems in chronic patients were “K86 K87 hypertension” (*n* = 237, 27.9%), “K74 K76 ischemic heart disease” (*n* = 199, 23.4%) and “T93 lipid metabolism disorder” (*n* = 153, 18.0%) (Table 3).

**Table 3:** The top 20 reasons for encounter of patients with symptoms and patients with chronic problems in descending order of frequency (classified based on the chapters in the ICPC- 2)^a^

Order	Symptoms	Frequency (n1=685, %)	Health problems in chronic patients	Frequency (n2=850, %)
1	R5 Cough	111 (16.2)	K86 K87 Hypertension	237 (27.9)
2	R21 Throat symptom/complaint	89 (13.0)	K74 K76 Ischaemic heart disease	199 (23.4)
3	R25 Sputum/phlegm abnormal	66 (9.6)	T93 Lipid disorder	153 (18.0)
4	R08 Nose symptom/complaint other	47 (6.9)	T89 T90 Diabetes	152 (17.9)
5	D12 Constipation	42 (6.1)	L95 Osteoporosis	57 (6.7)
6	P06 Sleep disturbance	41 (6.0)	Y85 Benign prostatic hypertrophy	14 (1.6)
7	R07 Sneezing/nasal congestion	22 (3.2)	T91 Vitamin/nutritional deficiency	13 (1.5)
8	D01, D02, D06 Abdominal pain	21 (3.1)	N94 Peripheral neuritis/neuropathy	9 (1.1)
9	F16 Eyelid symptom/complaint	20 (2.9)	R96 Asthma	5 (0.6)
10	L03 Low back symptom/complaint	19 (2.8)	K80 Cardiac arrhythmia NOS	4 (0.5)
11	D10 Vomiting	18 (2.6)	T92 Gout	2 (0.2)
12	S06 Rash localized	18 (2.6)	L86 Back syndrome with radiating pain	1 (0.1)
13	D19 Teeth/gum symptom/complaint	17 (2.5)	B80 Iron deficiency anaemia	1(0.1)
14	D08 Sneezing/nasal congestion	12 (1.8)	P76 Depressive disorder	1 (0.1)
15	U02 Urinary frequency/urgency	11 (1.6)	K96 Haemorrhoids	1 (0.1)
16	D03 Heartburn	10 (1.5)	D97 Liver disease NOS	1 (0.1)
17	N17 Vertigo/dizziness	10 (1.5)	-	-
18	L20 Joint symptom/complaint NOS	9 (1.3)	-	-
19	D09 Nausea	8 (1.2)	-	-
20	A03 Fever	8 (1.2)	-	-
	Others	86 (12.6)	-	-

Abbreviation: *ICPC- 2* International Classification of Primary Care, second edition

Note: ^a^there were 73 reasons for encounter of patients coming for test and therapeutic consultation

### Physical examinations provided by GPs

Among the 682 consultations, PE occurred in 108 (15.8%) consultations. In this study, there was statistically significant difference in PE between consultations with patients visiting with symptoms and without symptoms (*P* < 0.001). The performance of PE in consultations by GP subgroups showed no significant difference between consultations in CHCs and CHSs (15.8% in CHCs and 15.9% in CHSs, *P* > 0.05). PE was performed more frequently in consultations by female GPs (17.5% in female GPs’ consultations and 9.9% in male GPs’ consultations, *P* < 0.05). The performance of PE in consultations by GPs with different education experience (*P* > 0.05), professional positions (*P* > 0.05), working experience (*P* > 0.05), training experience (*P* > 0.05) showed no significant difference. GPs with medium amount (50-70) of patient visits was more likely to perform PE in consultations in comparison with GPs with smaller amount (<50) and larger amount (>70) of patient visits (*P* < 0.05) (Table 4).

**Table 4:** Frequency of physical examination in consultations by different GPs characteristics

Characteristics of GPs	Number of consultations	Consultations with PE (percentage, %)	Consultations with no PE (percentage, %)	χ2	P
Institution				0.000	0.994
CHC	518	82 (15.8)	436 (84.2)		
CHS	164	26 (15.9)	138 (84.1)		
Sex				5.069	0.024
Male	151	15 (9.9)	136 (90.1)		
Female	531	93 (17.5)	438 (82.5)		
Education				0.363	0.547
Bachelor’s degree	628	101 (16.1)	527 (83.9)		
Master’s degree	54	7 (13.0)	47 (87.0)		
Working years				1.813	0.178
≤10	147	18 (12.2)	129 (87.8)		
>10	535	90 (16.8)	445 (83.2)		
Professional positions				1.233	0.540
Junior grade title	88	11 (12.5)	77 (87.5)		
Intermediate grade title	268	41 (15.3)	227 (84.7)		
Senior grade title	326	56 (17.2)	270 (82.8)		
GP training				1.055	0.304
With training experience	490	82 (16.7)	408 (83.3)		
No training experience	192	26 (13.5)	166 (86.5)		
Patient visits				9.112	0.011
≤50	87	8 (9.2)	79 (90.8)		
51-70	360	71 (19.7)	289 (80.3)		
>70	235	29 (12.3)	206 (87.7)		
Total	682	108 (15.8)	574 (84.2)	-	-

Abbreviation: *GP* general practitioner, *PE* physical examination, *CHC* community health center, *CHS* community health station

Total 126 examinations were recorded in 108 consultations with PE. No patient was provided with comprehensive PE. The examinations performed by GPs were mainly distributed in “Head, face, and neck examination” (*n* = 54, 42.9%) and “Cardiovascular examination” (*n* = 55, 43.7%). PE was less performed in respiratory system (*n* = 7, 5.6%), musculoskeletal system (*n* = 8, 6.4%), and abdominal organs (*n* = 2, 1.6%). No PE was observed on the skin, male genitalia, female breasts and genitalia, and neurological system. Among 126 examinations, blood pressure measurement was most frequently observed (*n* = 49, 38.9%), followed by pharynx inspection (*n* = 43, 34.1%). The frequency of other PEs was less than 10 (Table 5).

**Table 5:** Frequency of physical examinations in 108 patient consultations (*n* = 126)

Components of PE	Frequency of PEs for patients coming with symptoms	Frequency of PEs for patients coming with chronic patients	Total (frequency, %)
General examination			
Temperature examination	0	0	0 (0.0)
Lymph node examination	0	0	0 (0.0)
Skin examination	0	0	0 (0.0)
Head, face, and neck examination			
Head and face inspection	1	0	1 (0.8)
Corneal and conjunctival examination	3	0	3 (2.4)
Pupillary light responses	1	0	1 (0.8)
Visual fields	1	0	1 (0.8)
Ophthalmoscopic examination	1	0	1 (0.8)
Mouth inspection	3	0	3 (2.4)
Pharynx inspection	43	0	43 (34.1)
Thyroid palpation	1	0	1 (0.8)
Cardiovascular examination			
Blood pressure measurement	16	31	49 (38.9)^a^
Pulse rate	0	1	1 (0.8)
Heart sounds oscultation	5	0	5 (4.0)
Respiratory examination			
Anterior chest percussion	2	0	2 (1.6)
Breath sounds oscultation of anterior chest	5	0	5 (4.0)
Abdominal examination			
Abdominal inspection	2	0	2 (1.6)
Abdominal palpation	0	0	0 (0.0)
Abdominal percussion	0	0	0 (0.0)
Auscultation of the abdomen	0	0	0 (0.0)
Musculoskeletal examination			
Lumbar percussion	1	0	1 (0.8)
Spine percussion	2	0	2 (1.6)
Inspection of hands	3	0	3 (2.4)
Inspection of lower limbs	2	0	2 (1.6)
Male genitalia examination	0	0	0 (0.0)
Female breasts and genitalia examination	0	0	0 (0.0)
Neurological examination	0	0	0 (0.0)
Total	92	32	126 (100.0)^a^

Abbreviation: *PE* physical examination

Note: ^a^there were 2 PEs of blood pressure measurement conducted for patients coming for a test

**Fig. 1:**
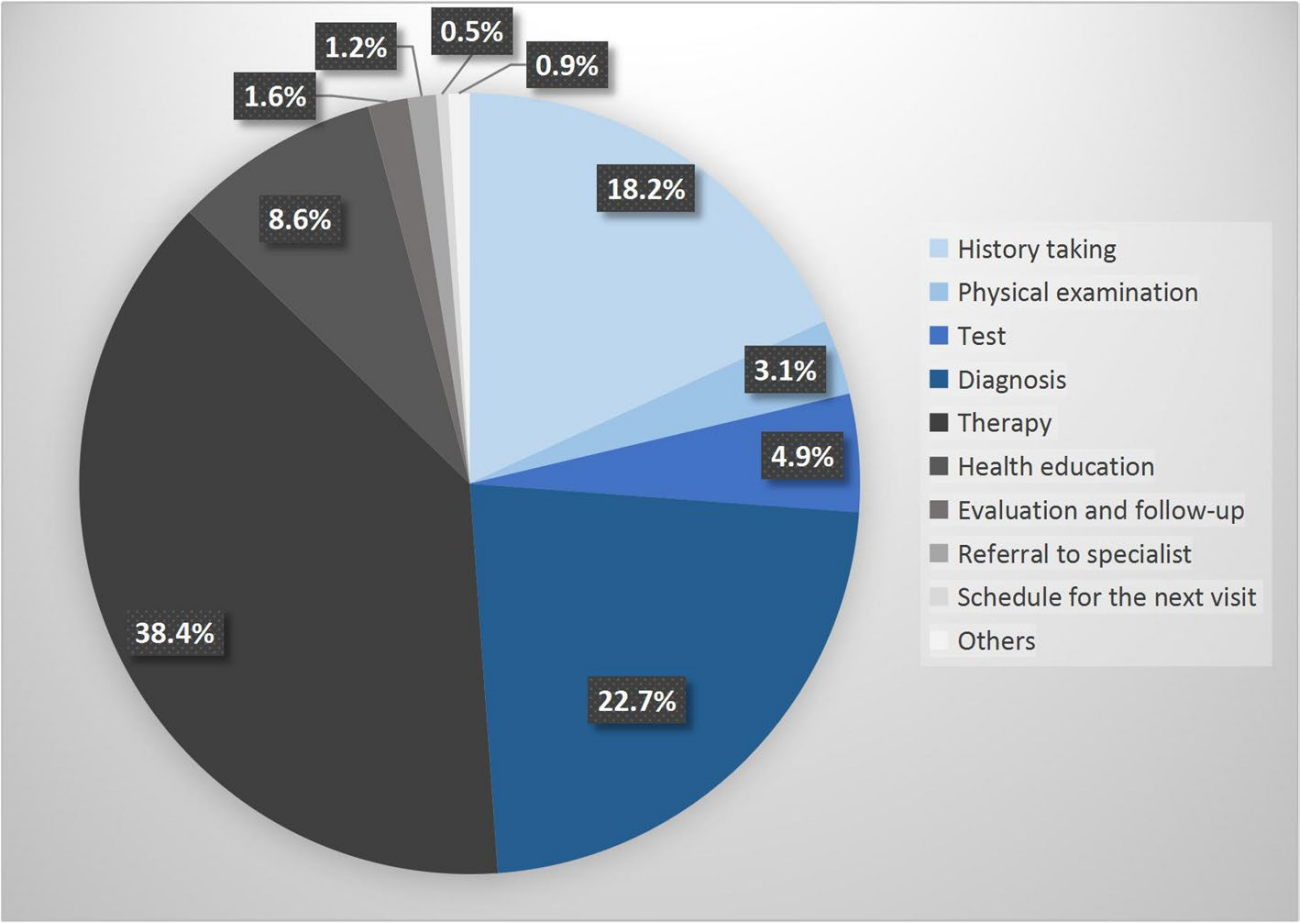
Time distribution of general practitioners’ activities in general practice consultations (*n* = 2823 minutes)

A total of 2615 minutes of general practice consultations were recorded. The mean length of consultation was 3.8±3.4 minutes (ranged from 1.0 to 37.0 minutes, *n* = 682). Because simultaneous activities were recorded, a total of 2823 minutes of activities were recorded. GPs only spent 3.1% of their time on PE, which was less than the time spent on taking history (18.2%), test (ordering tests or discussing test results with patients) (4.9%), and health education (8.6%). Over half of the time was spent on diagnosis (entering the diagnosis information into electronic medical record system or informing patients about the diagnosis) (22.7%) and therapy (providing therapy strategies or prescription to patients) (38.4%) (Fig. 1). The mean length of time spent in PE was 0.8±0.4 minutes (ranged from 0.5 to 2.0 minutes, *n* = 108).

## Discussion

### Main findings

In this study, we described GPs’ performance of PE in general practice consultations through direct observation in Beijing. The results showed that PE occurred in only 15.8% of general practice consultations. The frequency of PEs in each system varied, and the most frequent PE observed was blood pressure measurement (38.9%), followed by pharynx inspection (34.1%), which was consistent with the RFEs of patients with new symptoms and chronic health problems. In addition, GPs only spent 3.1% of the consultation time on PE, which was less than the time spent on taking history (18.2%), test (4.9%), diagnosis (22.7%), therapy (38.4%), and health education (8.6%).

### Comparisons with existing literature

The results obtained in this study demonstrated the insufficiency of PE in general practice consultations. Although PE was a fundamental skill of GPs in disease diagnosis and health promotion, it was performed by GPs in only 15.8% consultations in this study, which was even less than the results in a previous study in Beijing in 2013 (28.0%) [19]. Previous studies showed that PE was observed in 64.5% of general practice consultations in Australia (40 consultations by 4 primary care physicians, observed over 3 weeks) [7], 79.0% in Estonia (405 consultations, 15 consecutive patient visits to each family doctor) [17], and 72.8% in Guangzhou, China (445 consultations, 26 consultations per GP observed in one observation unit lasting for 3-4 hours) [18]. Comparing with findings in other countries and regions, the GPs in Beijing performed PE insufficiently in this study.

A lot of factors may influence the performance of PE by GPs. First, there may not be enough time for GPs to perform a complete examination. In the present study, the average length of consultation was 3.8±3.4 minutes. As indicated in a previous study, 5 min is necessary for physicians to take complete medical history and perform necessary PE [24]. In addition, GPs only spent 3.1% of their time on PE and the average time spent in PE was 0.8 minutes in this study, which was much shorter than the results in a study of Estonian family practices (2.0 minutes in PE) [17]. Comprehensive and appropriate PE for further investigation of elements about disease could lead to preventive care and health promotion counseling and would create opportunities for early diagnosis [25], in which sufficient time was undoubtedly necessary. Second, the reason of patient encounters may play an important role in the performance of PE. PE was more likely to be performed in patients with symptoms (*P* < 0.001). In Beijing, the patients with chronic diseases are usually managed in CHSIs, and medication refill on a monthly basis is accessible for chronic patients [26, 27]. In this study, most encounters in general practice clinics were chronic patients visiting for regular medication refill. PE was not necessarily needed for diagnosis, as information about disease is already in the electronic medical record system and most of the chronic patients were stable. Therefore, the GPs might consider it is not necessary to perform PEs for patients. Third, it cannot be ruled out that the clinical skills and professional competence of GPs in primary care still need to be improved [28]. In a survey covering 17 provinces in China, poor capacity and skills of the GPs were found to be the most common reasons for why patients bypassed primary care institutions when they needed clinical care (32%) [29].

In this study, the performance of PE in each system varied, with blood pressure measurement accounting for the most, followed by pharynx inspection. Blood pressure measurement is a standard procedure for GP in the follow-up of patients with chronic disease. However, the performance rate of blood pressure measurement is lower than that in a study from the US, in which all the patients (100.0%) received blood pressure measurement at their visits [30]. The finding of high frequency of pharynx inspection was similar with a study in Turkey, in which mouth and pharynx inspection occurred most frequently in general practice clinics [24]. In this study, the most common symptoms of patients were from respiratory system, including “R5 cough”, “R21 throat symptoms”, and “R25 sputum/sputum abnormalities”, which may be the cause of high frequency of pharynx inspection. Focused examination based on specific symptoms was most frequently performed, omitting those parts of examination believed to be low yield. However, previous evidence suggested the possibility that a simple way of strengthening the therapeutic alliance is to perform a few additional components of the PE at every visit, even in the absence of relevant symptoms, which may improve doctor–patient relationship [30].

In addition to the frequency of PEs performed by GPs, the extent of the inadequacies of the GPs in performing detailed items of the PE is worth-noticing. For patients visiting with symptoms, such as fever, localized rash, and abdominal pain, there was no examination of temperature, skin (asking patients to expose the skin and give an examination), and abdomen (palpation, percussion, auscultation of the abdomen) performed by GPs. Besides, although there were many patients visiting with symptoms in respiratory system (e.g. cough, sputum/phlegm abnormal), PE such as percussion and auscultation of the lungs were insufficiently performed by GPs. Oversight in PE could lead to missed or delayed diagnosis, which may be remedied if physicians paid more attention to PE in consultations [16].

Even for patients with chronic diseases, PE is very important for detecting complications. For instance, an analysis of SOLVD (Studies of Left Ventricular Dysfunction) showed that jugular venous distention (JVD) and a third heart sound (S3) were independently associated with progression of heart failure [31]. Patients with diabetes, several diabetic foot risk factors (neuropathy, foot deformity, minor trauma, previous ulceration or amputation) should be evaluated for lower extremity examination [32]. In this study, although most patients with hypertension, ischaemic heart disease, and diabetes were managed in CHSIs, the performance of PEs related to blood pressure, heart auscultation, and foot inspection and palpation was insufficient. PE is not only essential for diagnosis [8, 16, 33], but also critical in chronic disease management. Regular PE is one of the easiest, cheapest and most effective measure to prevent the complications of chronic diseases [34]. Therefore, more attention should be paid to PE for chronic patients in general practice.

### Strengths and limitations

Overall, the present study looked into the depth of PE performed in general practice consultations in Beijing. The results showed that PE was insufficiently performed by GPs in Beijing in terms of frequency and time. This may provide evidence for improving the quality of general practice consultations in CHSIs. In addition, the method itself is a strength, as the researchers were observing rather than relying on self-report.

Our study also has limitations. First, the generalizability of the findings is limited. For example, PE was less frequently performed by GPs with fewer patients (≤50 visits) than GPs with more patient visits in this study. This result may be inconsistent with a previous study [24], as fewer patient visits may lead to more time and chance for a PE. There were only 11 GPs recruited out of 29 GPs in 5 CHSIs in this study and only 2 GPs saw ≤ 50 patients, data trends may not be inferred. Besides, as indicated before, except for time constraint, RFEs also influence the performance of PE. And it is possible that the performance of PE varied in GPs with different work conditions and motivation. This is a preliminary study in exploration of GPs’ performance of PE, investigations in larger sample and analysis of influencing factors of PE are necessary in further researches. Second, the observer sitting in the consultation room may affect the GPs’ performance. GPs may behave better than usual when under observation. Therefore, we explained to the GP before the observation that this research would not affect their annual performance appraisal. During the observation, the observers were seated in the least intrusive corner of the consultation room to avoid disturbance. Third, observations might be influenced by observer bias and recording errors could be a possible limitation in data collection. We developed a structured observation form and modified it through a pilot study. We also provided careful training for observers about the principles of observation and information recording to ensure the consistency of observation. Finally, in this observational study, some activities for general examination (especially inspection, such as first impression, nutritional status, MSE) were not recorded due to the difficulty in identifying the examinations by observers. Therefore, the frequency of PEs performed by GPs might be underestimated.

### Implications for research and practice

This was a preliminary study showing the GPs’ performance of PE in GP-patient consultations, which can be used as a basis and reference for further research exploring the influencing factors and improvement strategies of PE performance in primary care. In addition, this study also provided feedback to GPs that there should be more focus on PE in GP-patient consultations. Moreover, it also provided an opportunity of future continuing medical education to improve the quality of medical services in primary care.

## Conclusion

PE was insufficiently performed by GPs in CHSIs in Beijing. More time and commitment should be advocated for appropriate PEs in primary care. Further researches may concentrate on exploration of the influencing factors of GPs’ performance of PE and improvement strategies of PE performance.

## Supplementary Information

**Additional file 1.**

## Data Availability

The datasets used and/or analyzed during the current study are available from the corresponding author on reasonable request.

## Abbreviations

PE: Physical examination
GPs: General Practitioners
CHSI: Community health service institution
CHC: Community health centre
CHS: Community health station
RFEs: Reasons for encounter
ICPC-2: International Classification of Primary Care second edition
SD: Standard deviation
SPSS: Statistical Package for Social Science
JVD: Jugular venous distention
S3: Third heart sound
