# Supplementary material for: Physical examination performed by general practitioners in 5 community health service institutions in Beijing: an observational study
Source: BMC Prim Care. 2022 Jan 14;23:7. doi: 10.1186/s12875-021-01619-1 (PMC8759261; doi:10.1186/s12875-021-01619-1)
Supplement: Supplementary file 1 — Additional file 1. [file 12875_2021_1619_MOESM1_ESM.docx]

| **NO.** |  |  |  |  |  |
| --- | --- | --- | --- | --- | --- |

**Observation form**

**1. Characteristics of Patients**

1.1 Sex：① Male ② Female

1.2 Age：_______ years

1.3 Consultation time：___________ — ___________ ；Length：_______ minutes

1.4 Medical insurance：

① Basic medical insurance

② Business insurance

③ No medical insurance

④ Other insurances, ______________

1.5 Reasons for encounter：

① Prescription for chronic disease (To 1.6)

② Come with symptoms (To 1.7)

③ Come for test/results (To 1.8)

④ Come for therapeutic consultation

⑤ Others, __________________________

1.6 The diagnosis/diagnoses of existing chronic disease(s) is/are __________________

1.7 The symptom(s)/complaint(s) is/are __________________

1.8 The diagnosis/diagnoses at this visit is/are __________________

**2.Medical activities performed by GPs**

| **Medical activities** | **Options** | **Time length** |
| --- | --- | --- |
| 2.1 History taking | ① Yes ② No |  |
| 2.2 Physical examination, which is/are_________________ | ① Yes ② No |  |
| 2.3 Test (order for tests and discuss test results) | ① Yes ② No |  |
| 2.4 Make a diagnosis (inform patients verbally and enter diagnostic information into the electronic medical record system) | ① Yes ② No |  |
| 2.5 Therapy (prescription, therapeutic counselling, wound care, bandage, et.al) | ① Yes ② No |  |
| 2.6 Health education | ① Yes ② No |  |
| 2.7 Evaluation and follow-up | ① Yes ② No |  |
| 2.8 Referral to specialist | ① Yes ② No |  |
| 2.9 Schedule for the next visit | ① Yes ② No |  |
| 2.10 Others, _________________ | ① Yes ② No |  |
